# 28-day perioperative pediatric mortality and its predictors in a tertiary teaching hospital in Ethiopia: a prospective cohort study

**DOI:** 10.1186/s40001-023-01613-6

**Published:** 2024-01-05

**Authors:** Misganew Terefe Molla, Nebiyu Shitaye Anley, Bekalu Wubshet Zewdie, Amanuel Sisay Endeshaw, Fantahun Tarekegn Kumie

**Affiliations:** 1 Department of Anesthesia, College of Medicine and Health Sciences, Bahir Dar University, Bahir Dar, Ethiopia; 2 Department of Surgery, Pediatric Surgery Unit, College of Medicine and Health Sciences, Bahir Dar University, Bahir Dar, Ethiopia; 3 Department of Orthopedics and Traumatology, Pediatric Orthopedic Unit, College of Medicine and Health Sciences, Bahir Dar University, Bahir Dar, Ethiopia

**Keywords:** Pediatric, Mortality, Perioperative, Surgery, Ethiopia

## Abstract

**Background:**

Perioperative pediatric mortality is significantly higher in low-resource countries due to a scarcity of well-trained professionals and a lack of well-equipped pediatric perioperative services. There has been little research on pediatric mortality in low-income countries. Therefore, this study aimed to assess the incidence of perioperative pediatric mortality and its predictors in 28-day follow-up.

**Methods:**

The data were collected using REDCap, an electronic data collection tool, between June 01, 2019 and July 01, 2021. This study includes pediatric patients aged 0 to 17 years who underwent surgery in Tibebe Ghion Specialized Hospital over 28 days with a total of 1171 patients. STATA version 17 software was used for data analysis. Log-rank tests were fitted to explore survival differences. After bivariable and multivariable Cox regression analysis, an Adjusted Hazard Ratio (AHR) with a 95% Confidence Interval (CI) was reported to declare the strength of association and statistical significance.

**Results:**

There were 35 deaths in the cohort of 1171 pediatric patients. Twenty of the deaths were in neonates. The overall perioperative mortality among pediatric patients was 2.99%, with an incidence rate of 1.11 deaths per 1000 person day observation (95% CI 0.79, 1.54). The neonatal age group had an AHR = 9.59, 95% CI 3.77, 24.3), transfusion had an AHR = 2.6, 95% CI 1.11, 6.09), and the America Society of Anesthesiology physical status classification III and above had an AHR = 4.39, 95% CI 1.61, 11.9 were found the significant predictors of perioperative pediatric mortality.

**Conclusions:**

In this study, the perioperative mortality of pediatric patients was high in the 28-day follow-up. Neonatal age, transfusion, and America Society of Anesthesiology physical status III and above were significant predictors of pediatric mortality. Therefore, perioperative surgical teams should give special attention to neonates, the America Society of Anesthesiology physical status III and above, and transfusion to reduce pediatric mortality.

**Supplementary Information:**

The online version contains supplementary material available at 10.1186/s40001-023-01613-6.

## Introduction

More than 2 billion people generally require emergency and necessary surgical treatments, and up to 11% of illnesses that fall under the category of surgical conditions require surgical intervention [1]. Estimated 234 million surgical procedures are performed annually, and over 75% of all surgical procedures coverage was in high-income countries. However, in low- and middle-income countries (LMICs), particularly in sub-Saharan Africa, the surgical coverage was around 4% [2]. In sub-Saharan Africa, especially in patients from distant, violent locations, the proportion of pediatric hospitalized patients who need surgery may range from 6 to 12% [3]. Children have different surgical needs than adults, and they have more difficult perioperative anesthetic issues because they undergo surgery and anesthesia at a crucial time in their development [3].

In LMICs, perioperative mortality related to surgery, anesthesia, the disease entity itself, or complications in the postoperative period is high. However, the magnitude of the LMICs was more significant than anticipated because of poor infrastructure, a shortage of pediatric surgeons and anesthesia providers, and a lack of access to surgical and anesthesia equipment for their service delivery [1, 3–5]. Inadequate facilities, delivery methods, and labor availability are the main reasons why the health systems and surgical care for children in Africa, in particular, fall behind those in the rest of the world. The other major factors are the lack of health insurance, the high cost of medical equipment, and the rising price of pharmaceuticals [6, 7].

In LMICs, perioperative pediatric mortality is higher than in high-income countries; it can reach up to 54% in various surgical procedures as a result of the delayed presentation, poor facilities, untrained staff, a lack of intensive care units, and a lack of use of the safe operations checklist [8, 9]. The health strategy in LMICs for pediatric mortality in the perioperative period does not provide special attention because insufficient data demonstrate the full scope and severity of the problem, and is not viewed as a public health priority [3].

Perioperative pediatric mortality in New York revealed that the mortality rate within 30 days of follow-up was 0.3% to 3.6% [10]. A study in Nigeria revealed that the mortality rate was 3.2% within 30 days of follow-ups [11]. A Kenyan study shows that 1.7% of death recorded on the seventh postoperative day [9], and a study conducted at Tibebe Ghion Specialty Hospital (TGSH) in Ethiopia was 2.58% on the seventh-day follow-up [12]. Factors including emergency surgery, neonatal age, the American Society of Anesthesiology (ASA) class III and above, congenital anomalies, multiple surgical procedures, night or weekend surgery, and the lack of a surgical safety checklist that contributes to perioperative mortality in pediatric populations were identified [9, 10, 13].

There is a limited study on pediatric perioperative outcomes in LMICs with large sample sizes, even though it is a critical quality indicator in perioperative patient outcomes. No published study in Ethiopia shows the overall perioperative pediatric mortality and its predictors on a 28-day follow-up. Therefore, this study aimed to assess the incidence and predictors of perioperative pediatric mortality in 28-day follow-up.

## Methods

### Study design, setting, and period

It is a prospective cohort study conducted from June 01, 2019 to July 01, 2021. The study setting was in Northwest Ethiopia, Bahir Dar, which was 580 km from Addis Ababa, the capital city of Ethiopia.

The hospital has more than 500 beds and 14 major operation theaters for emergency and elective surgeries. TGSH is a tertiary-level hospital that serves over 5 million populations provided in various units with specialities and subspecialist medical doctors, including general surgery, orthopedic, obstetrics, gynecology, head and neck, ear, nose, and throat, pediatric, hepatobiliary, maxillofacial, cardiothoracic, gastrointestinal, urological, and neurologic surgery. Additionally, it houses around 1000 healthcare professionals.

This study was conducted in line with the Strengthening the Reporting of Observational Studies in Epidemiology (STROBE) statement: guidelines for reporting observational studies [14] (see Additional file 1).

### Eligibility criteria

This study covered all pediatric patients under 18 years who underwent surgery at TGSH between June 01, 2019 and July 01, 2021. Patients without cell phones were not included in this study.

### Variables of the study

#### Dependent variable

Time to death until 28-day perioperative day.

#### Independent variables

Age, gender, types of surgery, ASA status, coexisting disease, the urgency of surgery, surgical safety checklist, transfusion, and anesthesia type.

### Data collection methods and quality control

The Research Electronic Data Capture (REDCap) platform, an offline data input system in which the anesthetist collects the data based on uploaded questionnaires in the system, served as the foundation for the data gathering techniques.

The anesthetist obtained the perioperative data for up to 28 days and was the study’s primary data collector. For inpatient mortality, the data manager monitored the data's accuracy and double-checked it against the hospital logbook system. However, the patient condition was gathered over the phone for patients discharged from the hospital to assess the outcome during the 28-day follow-up. We have obtained multiple phone numbers from family members in order to reach patients for follow-up and decrease the dropout rate.

The REDCap database system is under the control of the information technologist. Before collecting the data, the anesthetist received training on the data collection method and how to keep patient records. The acquired data were stored in the REDCap database system to guarantee data quality, only available to authorized individuals.

### Data management and analysis

STATA Version 17 software was utilized to carry out the data analysis procedures. Descriptive statistics describe demographic and clinical characteristics. We calculated the hazard ratio in the bivariable analysis with a 95% confidence interval (CI), and variables with a *P*-value of < 0.2 were considered in the multivariable analysis. The AHR with a 95% CI was calculated in the multivariable analysis, and *P*-value < 0.05 were considered statistically significant predictors.

Using a log-rank test, the survival curves of different study subject groups were compared. We examined the Cox proportional hazard assumption before building a regression model (log–log plot). To determine risk factors for postoperative mortality, parametric and semi-parametric survival analysis models were fitted. Missing data were analyzed by using available case analysis.

### Ethical statement

The Institutional Review Board of Bahir Dar University, College of Medicine and Health Sciences, granted ethical approval to conduct this study (reference number CMHS/IRB 0163/18). A waiver of informed consent was obtained prior to data collection. All the information obtained from the patients was kept confidential and secure from unauthorized access.

## Results

### Demographic characteristics of pediatric surgical patients

**Fig. 1:**
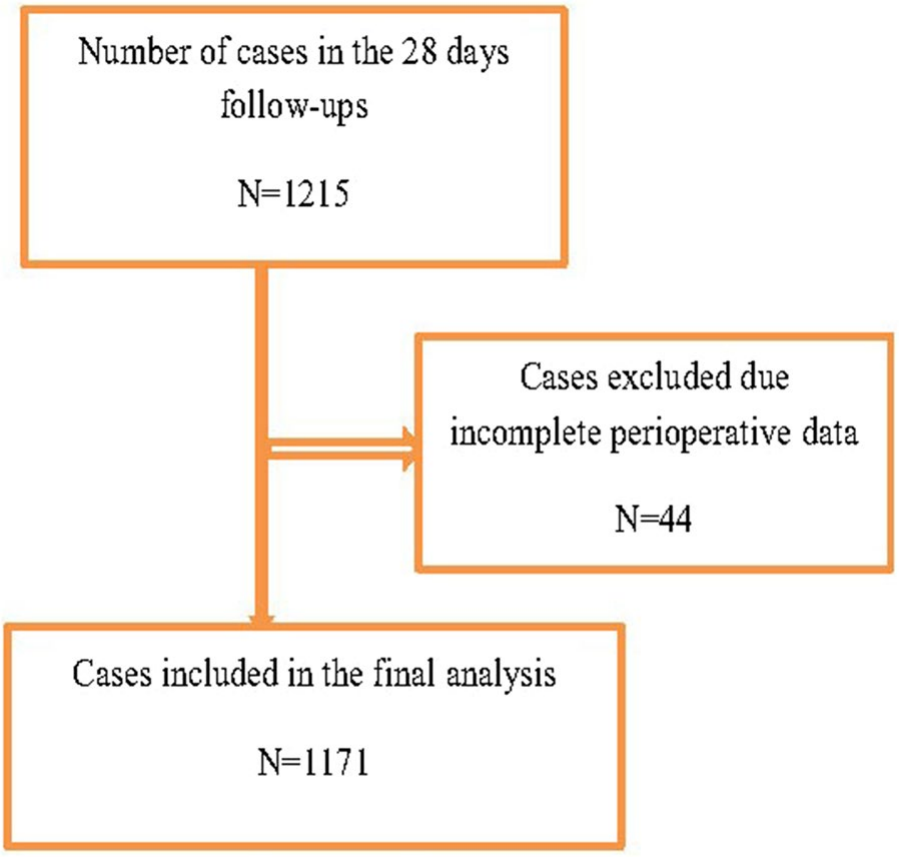
Study population profile

In this study, 1171 patients enrolled who underwent surgical intervention from June 1, 2019, to September 30, 2022, with a response rate of 95.7% (Fig. 1). From 1171 total cases, 50.1% were age ranges from 5 to 17 years, 50.1%. Most pediatric patients were male, 66.5% (Table 1).

**Table 1:** Demographic characteristics of pediatric surgical patients in TGSH Northwest Ethiopia, 2023

Variables	Categories	Total (*N* = 1171)	Outcome	
			Survived *(N* = 1136)	Death (*N* = 35)
Age	≤ 30 days	173 (14.8%)	153 (88.4%)	20 (11.6%)
	1–12 months	192 (16.4%)	188 (97.9%)	4 (2.1%)
	1–5 years	219 (18.7%)	216 (98.6%)	3 (1.4%)
	5–17 years	587 (50.1%)	579 (98.6%)	8 (1.4%)
Sex	Male	779 (66.5%)	752 (96.5%)	27 (3.5%)
	Female	392 (33.5%)	384 (98%)	8 (2%)

### Clinical characteristics of pediatric surgical patients

In this study, out of 1171 children who underwent surgery, general surgery accounted for 39.2% of the operations. Most surgeries were performed under emergency conditions 53.3%. The majority of cases, 74.6%, were non-trauma. In about 88.1% of cases, there is no comorbid illness, and 95.5% of those cases had ASA physical statuses of one or two. In our study, 89.3% of the cases did not require transfusion, and 95.5% of the patients began with the WHO surgical safety checklist completed (Table 2).

**Table 2:** Clinical characteristics of pediatric surgical patients in TGSH Northwest Ethiopia, 2023

Variables	Categories	Total (N = 1171)	Outcome	
			Survived (N = 1136)	Death (N = 35)
Procedure type	General surgery	459 (39.2%	442 (96.3%)	17 (3.7%)
	Orthopedics	312 (26.6%	310 (99.4%)	2 (0.6%)
	Neurosurgery	193 (16.5%)	178 (92.2%)	15 (7.8%)
	ENT	127 (10.9%)	126 (99.2%)	1 (0.8%)
	Urology	60 (5.1%)	60 (100%)	0
	Endocrine	6 (0.5%)	6 (100%)	0
	Other	14 (1.2%)	14 (100%)	0
Urgency of surgery	Elective	547 (46.7%)	533 (97.4%)	14 (2.6%)
	Emergency	624 (53.3%)	603 (96.6%)	21 (3.4%)
Anesthetic type	General anesthesia	982 (83.9%	948 (96.5%)	34 (3.5%)
	Regional anesthesia	189 (16.1%)	188 (99.5%)	1 (0.5%)
Trauma	Yes	297 (25.4%)	288 (97%)	9 (3%)
	No	874 (74.6%)	848 (97%%)	26 (3%)
Coexisting disease	Yes	139 (11.9%)	132 (95%)	7 (5%)
	No	1032 (88.1%)	1004 (97.3%)	28 (2.7%)
WHO safety checklist	Yes	1118 (95.5%)	1084 (97%)	34 (3%)
	No	53 (4.5%)	52 (98.1%)	1 (1.9%)
Transfusion	Yes	125 (10.7%)	117 (93.6%)	8 (6.4%)
	No	1046 (89.3%)	1019 (97.4%)	27 (2.6%)
ASA physical status classification	ASA I and II	1116 (95.3%)	1089 (97.4%)	27 (2.6%0
	ASA III, IV and V	55 (4.7%)	47 (85.5%)	8 (14.5%)

WHO world health organization; ASA America society of anesthesiology

### 28-day perioperative pediatric mortality

**Fig. 2:**
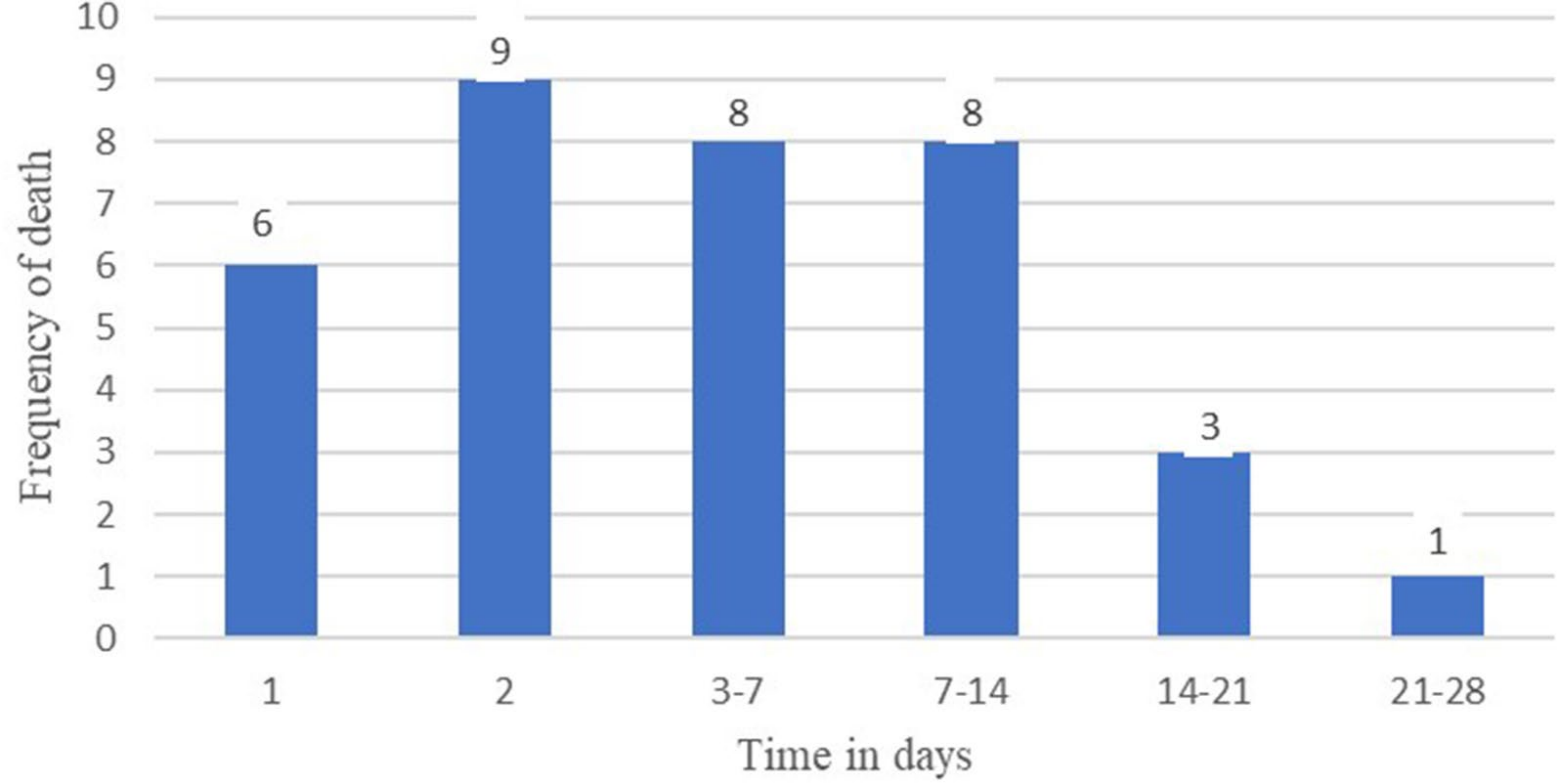
Frequency of deaths in each postoperative time interval

In the 28-day follow-up, 35 deaths were recorded among 1171 pediatric surgical patients. The most common cause of death among these procedures was intra-abdominal surgery (17 patients) or 3.3% of the procedure was died, followed by a ventriculoperitoneal shunt (VP-shunt) was 12 patients or 8.5% of patients was died (Table 3). Pediatric mortality in the 28-day follow-up was high at two days of postoperative period (Fig. 2).

**Table 3:** Description of patients who died based on their procedure in TGSH Northwest Ethiopia, 2023

Specific procedure type	Total (*N* = 1171)	Number of deaths (*N* = 35)	Proportion of deaths
General surgery, and Urology procedure	519	17	3.3
Meningomyelocele repair	52	3	5.8
Ventriculoperitoneal shunt	141	12	8.5
Upper extremity procedure	75	0	0
Lower extremity procedure	237	2	0.8
Ear, nose, and throat procedure	127	1	0.8
Other surgical procedure	20	0	0

### Predictors of perioperative pediatric mortality

In the multivariable analysis, age, transfusion, and ASA physical status were significantly associated with pediatric mortality. The hazard of perioperative mortality among neonates was 9.59 (AHR = 9.59; 95% CI 3.77, 24.3) times higher than infants and above-age groups. Pediatrics with ASA physical status classification III/IV/V have 4.39 (AHR = 4.39; 95% CI 1.61, 11.9) times increased hazard of perioperative death compared to their counterparts. The risk of perioperative mortality was 2.6 (AHR = 2.6; 95% CI 1.11, 6.09) times higher among pediatrics who were transfused with blood than those who did not (Table 4).

**Table 4:** Bivariable and multivariable Cox proportional hazard regression analysis of predictors of perioperative pediatric mortality in TGSH Northwest Ethiopia, 2023

Variable	Categories	Total (*N* = 1171)	Death	CHR(95% CI)	AHR(95% CI)
Age	≤ 30 days	173	20	8.81 (3.88, 20.0)	9.59 (3.77, 24.3)**
	1–12 months	192	4	1.53 (0.46, 5.07)	1.89 (0.51, 7.04)
	1–5 years	219	3	1.01 (0.27, 3.79)	1.14 (0.29, 4.47)
	5–17 years	587	8	1	1
Anesthetic type	General	982	34	6.61 (0.9, 48.3)	3.2 (0.41, 25.3)
	Regional	189	1	1.00	1.00
Trauma	Yes	277	9	1.09 (0.51, 2.34)	1.94 (0.78, 4.83)
	No	894	26	1	1
Coexisting disease	Yes	139	7	1.85 (0.81, 4.25)	0.69 (0.24, 1.97)
	No	1032	28	1	1
WHO safety checklist	Yes	1118	34	1.51 (0.21, 11.0)	1.38 (0.18, 10.4)
	No	53	1	1	1
Transfusion	Yes	125	8	2.51 (1.14, 5.52)	2.6 (1.11, 6.09)*
	No	1046	27	1	1
ASA physical status classification	I/II	1116	27	1	1
	III/IV/V	55	8	4.46 (1.85, 10.7)	4.39 (1.61, 11.9)*

AHR, adjusted hazard ratio; CHR, crude hazard ratio; CI, confidence interval; WHO, world health organization; ASA, America society of anesthesiology

^*^p-value < 0.05, **p-value < 0.001

## Discussion

The primary purpose of this study was to investigate the incidence and predictors of 28-day perioperative pediatric mortality in a tertiary teaching hospital in Ethiopia. Most published data regarding pediatric perioperative mortality are from high-income countries where a considerable difference exists in the level of health facilities and medical personnel. Therefore, this study used a prospective data collection tool to investigate demographic and clinical variables in a mixed pediatric surgical cohort.

In our study, the overall perioperative pediatric mortality in the 28 days was 2.99% with an incidence rate of 1.11 deaths per 1000 person day observation which was slightly higher than the previous study from the same institution, which revealed 2.58% mortality [12]. The possible explanation for this discrepancy could be a difference in the sample size and duration of follow-up. Our result shows that perioperative pediatric mortality was high compared to a study conducted in high-income countries, which ranged between 0.7% and 1.1% [15, 16]. The poor surgical and anesthesia service infrastructure, scarcity of well-organized intensive care setups, and scarcity of well-trained pediatric surgical teams were the possible reasons for this higher result [17–19]. Contrarily our result was lower than a pediatric surgical outcome report of 9.9% mortality conducted in 19 sub-Saharan African countries [20]. The possible explanation might be in setting the availability of sub-speciality pediatric surgeons and senior master anesthetists for handling pediatric surgery and the increment in resource provision for pediatric surgery.

This study found that neonates had a nearly tenfold risk of postoperative pediatric deaths compared to infant and above-age groups. This finding was supported by a study conducted in Nigeria [11, 13]. Shreds of evidence suggest that the mortality of neonates was due to different congenital abnormalities, birth at younger gestational age, and complication of prematurity such as infection and sepsis [21–23].

Pediatrics with ASA physical status classification III/IV/V had an increased risk of perioperative death compared to pediatrics with ASA physical status classification I/II. This finding was in line with studies conducted in Kenya [9], the United States of America [24], Nigeria [13, 25], and Brazil [26]. An explanation for this link might be that patients with increasing ASA physical status classification have a poor physiologic reserve to tolerate stressful conditions of surgery and anesthesia. Additionally, a higher ASA physical status classification indicates a severe disease process that increases the risk of perioperative mortality [27–29].

As evidenced by the results of previous studies [30, 31], blood transfusion was a significant predictor of perioperative death among pediatrics in this study. The possible explanation could be that transfusion increases the risk of transfusion-related complications and immunosuppression, which increases the risk of infection, organ dysfunction, and overall morbidity and mortality [32, 33].

The clinical importance of this study was to provide information about risk factors associated with perioperative mortality among pediatrics and to act on minimizing the risk of perioperative death. As a strength, this study used a prospective data collection tool, a long follow-up period, and a survival analysis model, which helps better understand the predictors of perioperative death among children. Nonetheless, it has limitations, such as being a single-center study with a small sample size.

## Conclusion

In this study, perioperative pediatric mortality is high in 28 days of follow-up. Those transfused patients, ASA physical status classification III and above, and neonatal age were independent predictors of perioperative pediatric mortality.

### Supplementary Information

**Additional file 1.** STROBE checklist.

## Data Availability

Data used to generate the result of this study are available from the corresponding author upon reasonable request.

## Abbreviations

AHR: Adjusted Hazard Ratio
ASA: America Society of Anesthesiology
CHR: Crude Hazard Ratio
CI: Confidence Interval
LMICs: Low- and Middle-Income Countries
TGSH: Tibebe Ghion Specialized Hospital
WHO: World Health Organization
